# Effects of Simulated Nitrogen Deposition on Soil Respiration in a *Populus euphratica* Community in the Ebinur Lake Area, a Desert Ecosystem of Northwestern China

**DOI:** 10.1371/journal.pone.0137827

**Published:** 2015-09-17

**Authors:** Xuemin He, Guanghui Lv, Lu Qin, Shunli Chang, Min Yang, Jianjun Yang, Xiaodong Yang

**Affiliations:** 1 Xinjiang Key Laboratory of Oasis Ecology, Xinjiang University, Urumqi, Xinjiang, China; 2 College of Resources and Environment Science, Xinjiang University, Urumqi, Xinjiang, China; 3 Xinjiang Academy of Environmental Protection Science, Xinjiang Department of Environmental Protection, Urumqi, Xinjiang, China; DOE Pacific Northwest National Laboratory, UNITED STATES

## Abstract

One of the primary limiting factors for biological activities in desert ecosystems is nitrogen (N). This study therefore examined the effects of N and investigated the responses of an arid ecosystem to global change. We selected the typical desert plant *Populus euphratica* in a desert ecosystem in the Ebinur Lake area to evaluate the effects of N deposition on desert soil respiration. Three levels of N deposition (0, 37.5 and 112.5 kg·N·ha^-1^·yr^-1^) were randomly artificially provided to simulate natural N deposition. Changes in the soil respiration rates were measured from July to September in both 2010 and 2013, after N deposition in April 2010. The different levels of N deposition affected the total soil N, soil organic matter, soil C/N ratio, microorganism number, and microbial community structure and function. However, variable effects were observed over time in relation to changes in the magnitude of N deposition. Simulated high N deposition significantly reduced the soil respiration rate by approximately 23.6±2.5% (P<0.05), whereas low N deposition significantly increased the soil respiration rate by approximately 66.7±2.7% (P<0.05). These differences were clearer in the final growth stage (September). The different levels of N deposition had little effect on soil moisture, whereas N deposition significantly increased the soil temperature in the 0–5 cm layer (P<0.05). These results suggest that in the desert ecosystem of the Ebinur Lake area, N deposition indirectly changes the soil respiration rate by altering soil properties.

## Introduction

Increased nitrogen (N) deposition changes the structure and function of ecosystems and causes a series of ecological effects [1,2]. With the intensification of human activities in recent years, the ecological effects of N deposition have gained significant public attention [3–9]. The globalization of economic development has been accompanied by increased N deposition worldwide [10], and a large amount of anthropogenic nitrogen is constantly being incorporated into terrestrial and aquatic ecosystems, thereby affecting the global biogeochemical cycle [11–14]. However, the potential responses of soil carbon (C) dynamics to the rapidly increasing global active N pool are unclear [15,16]. Increased N deposition may accelerate or decelerate the release of C in humus, thus affecting the relationship between N deposition and C sequestration in temperate forest ecosystems [17–19]. The effects of simulated N deposition on soil respiration have been the focus of an increasing amount of research, much of which has focused on forest ecosystems [2,20–23]. Although reports on this topic have been varied, they show that low levels of N deposition have the potential to promote soil C release processes [24,25], whereas high levels of N deposition may inhibit these processes [1,26,27].

N affects respiration directly through several mechanisms, and the energy produced by respiration can be used to support root absorption and N assimilation [28]. N also indirectly affects soil respiration by affecting ecosystem production [29]. For example, Vitousek and Howarth [30] found that adding N could stimulate plant primary production by supplying a larger amount of substrate for soil respiration. However, adding N may reduce the root-to-stem ratio and lead to a decrease in soil respiration [31].

Arid and semiarid ecosystems receive limited water and N [32]. Therefore, studying the effects of N deposition on soil respiration is vitally important for improving our understanding of the response of arid ecosystem C cycles to global change. Soil N content is lower in arid and semiarid regions under natural conditions; therefore, a small amount of N can alleviate soil N limitations and produce larger ecological effects in arid and semi-arid areas. As reported by Baez et al. [33], N deposition in the northern Chihuahuan Desert can increase the local herb coverage, reduce leguminous plant abundance and native plant dominance, and change the plant community structure. Zhou et al. [34] analyzed the growth and photosynthetic physiological responses of two annual plants to N and water and showed that springtime N and water fluctuations in this desert are beneficial to the growth and productivity of *M*. *africana* and *B*. *hyssopifolia*, especially the aboveground parts of the plants. Plant growth is extremely important for the regulation of CO_2_ flux in response to N deposition. Yan et al. [35] showed that N deposition significantly increased the net ecosystem exchange (NEE) during the growing seasons in a temperate semiarid steppe in Duolun County. Similarly, Chen et al. [36] conducted research on degraded semiarid grasslands of Inner Mongolia in China and found that water and N deposition could significantly promote ecosystem respiration by 47–70% (P < 0.01). However, soil respiration in response to N is rarely reported for arid desert forest ecosystems with high-intensity light and dry conditions.

Xinjiang is composed of arid and semi-arid regions, and the amount of applied N fertilizer in the area has increased rapidly with aggressive agricultural development, from 7.4×10^7^ kg·a^-1^ in 1980 to 4.9×10^8^ kg·a^-1^ in 2004 [34,37]. The study area is a desert ecosystem connected to an oasis farmland. A large amount of N has spread from the farmland into the nearby desert ecosystem through volatilization, run off and other mechanisms. Most of the N deposited to the soil is in the form of wet deposition, which may cause N pulses when the snow melts in the spring and partially alleviate the impact of summer drought on local plants [34].

In this paper, we performed a controlled experiment testing the effects of varying magnitudes of N deposition on soil respiration within communities of a typical desert ecosystem plant, *Populus euphratica*. We also investigated environmental factors that affect the soil respiration rate. These experiments were designed to answer the following questions: (1) How do different levels of N deposition affect soil physical and chemical properties, and how do these effects change over time? (2) How do different levels of N deposition affect the respiration of *P*. *euphratica* community soils, and how do these effects change with time? (3) Is temperature the environmental factor with the greatest effect on soil respiration rate? How does the sensitivity of soil respiration to temperature vary with the level of N deposition, and does the time since deposition affect this sensitivity? This research will improve our understanding of the effects of increased N deposition on desert ecosystems and provide a theoretical foundation for the management of desert ecosystems.

### Materials and Methods

#### Study area description

The study area is located in the northwest periphery of the Junggar Basin (82°36′-83°50E, 44°30′-45°09′N) within the Xinjiang Uygur autonomous region. This region covers an area of 2670.85 km^2^; it has a soil N quantity of 0.255 g/kg, and the N deposition rate is 3.012 kg·N·ha^-1^·yr^-1^ (for reference [38], the average value for the Xinjiang Province was 3.012 kg·N·ha^-1^·yr^-1^). Consistent with a typical continental climate, this region is extremely dry and windy and has scarce rainfall and frequent dust storms. Winters and summers are long, whereas springs and autumns are short. The mean annual temperature, precipitation and potential evapotranspiration are 7.7°C, 102 mm and 1447 mm, respectively [39]. *P*. *euphratica* is a typical halophyte riparian forest desert plant that commonly grows within the study area.

#### Experimental design and ethics statement

The experiments were conducted at the East Bridge management station (44°37′N, 83°34′E) in the Nature Reserve of the Ebinur Lake Wetland. For the study objects, we selected nine *P*. *euphratica* plants with similar growth from soils with similar characteristics. Because the levels of soil N and N deposition in our study area were less than those of other forest ecosystems, congeneric study areas and congeneric international research methods [26,40,41], we decreased the intensity and frequency of N deposition in this experimental treatment. In this experiment, we set three N concentration treatments as follows: control (0 kg·N·ha^-1^·yr^-1^, CK), low-N (L, 37.5 kg·N·ha^-1^·yr^-1^), and high-N (H, 112.5 kg·N·ha^-1^·yr^-1^). We randomly chose three *P*. *euphratica* individuals for each treatment. At the end of April in 2010, we defined a sample plot of approximately 2 m×2 m around each *P*. *euphratica* plant, dug a 30-cm-deep ditch around the tree and added the required weight of CO(NH_2_)_2_. We used a portable sprayer to uniformly distribute a liter of water over each sample plot. The control groups received an equal quantity of water.

No specific permission was required for the described field studies in the Ebinur Lake Wetland. This area is owned and managed by the local government, and the location, including the site used for our experiment, is not privately owned or protected. Thus, specific permits were not required for non-profit research. Finally, the field studies did not involve endangered or protected plant species.

### Research Methods

#### Soil respiration rate measurement

**Fig 1:**
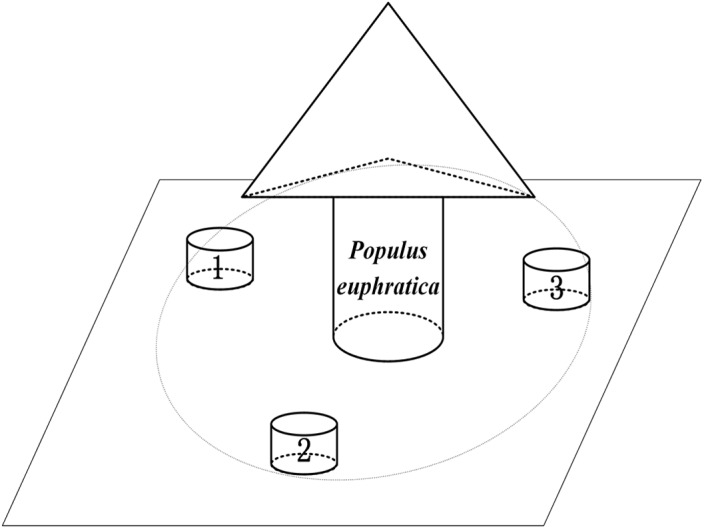
Representation of the quadrats of soil collars under the canopy of each *P. euphratica* in the studies Each treatment contained three plots (one *P. euphratica* in one plot), each plot consisted of three subplots, three subplots distribute the east, south and west around the *P. euphratica*, for a total of nine replicates in one site.

Soil respiration was measured in each sample plot with a flat and uniform terrain using an automated CO_2_ efflux system (LI-8100, LI-COR Inc., Lincoln, NE, USA). Three replicate measurements were recorded for each sample plot (Fig 1). During the study period, three polyvinyl chloride (PVC) collars (20 cm inner diameter, 10 cm height) were inserted 7 cm into the soil of each plot and left in the same locations throughout the study. Living plants inside the soil collars were clipped as close to the soil surface as possible without disturbing the surface litter.

After a 24-hour equilibration period, the soil respiration rate reverted to its base level before the PVC was placed. Measurements were recorded after this 24-hour period to avoid short-term respiratory rate fluctuations caused by disturbances from placement of the gas chamber (for raw data regarding the soil respiration rate, see S1 Appendix).

#### Measurement time

Soil respiration was measured on the 20^th^ and 30^th^ of July and on the 20^th^ of September in 2010 and 2013. We also selected several days with stable weather during which continuous daytime (local time between 7:00 and 19:00 at 2-hour intervals) and nighttime (local time between 22:00 and 4:00 the next morning at 3-hour intervals) soil respiration measurements were performed. Furthermore, several days with stable weather close to July 20^th^ and September 20^th^ in 2013 were selected for hourly soil respiration measurements throughout the entire day.

#### Measurements of environmental factors

Concurrently with the soil respiration rate measurements, the air temperature, atmospheric relative humidity and wind speed were measured at 150 cm and 10 cm above the ground using a handheld weather instrument (Kestrel 3000, USA). Additionally, the soil temperature at depths of 5, 10, 15, 20 and 25 cm was measured using two geothermometers (raw data regarding environmental factors are provided in S1 Appendix).

#### Soil sample collection and processing

At each site, three soil cores were collected to a depth of 50 cm with a hole beyond the observation point of soil respiration. Core measurements were performed on the 0–5, 5–15, 15–30, and 30–50 cm depth sections. Soil samples were collected following the three-point method, the litter and impurities were removed, and then the samples were stored in sealed bags until the soil properties were determined. The total nitrogen (TN) was measured following the Kjeldahl procedure (UDK140 Automatic Steam Distilling Unit, Automatic Titroline 96, Italy), soil organic matter was measured following the K_2_Cr_2_O_7_–H_2_SO_4_ oxidation method, and soil moisture was determined following the drying method [42]. Microbial quantities were determined using the dilution plate method [43], and three types of microorganisms were observed: bacteria, fungi, and actinomycetes (for raw data regarding TN, soil organic matter, soil moisture and microorganism quantity, see S1 Appendix).

### Statistical Analysis

The sensitivity coefficient of soil respiration in response to temperature was calculated using the following formulae:

$$Rs=ae^{bt}$$

$$Q_{10}=e^{10b},$$

where Rs is the measured respiration rate (μmol·m^-2^·s^-1^), T is the soil temperature (°C), and a and b are the regression coefficients. An exponential curve regression was performed on the soil respiration rate and corresponding temperature data from July to September in 2010 and 2013.

A one-way analysis of variance (ANOVA) was performed for the data, and Fisher’s least significant difference (LSD) method was used to conduct multiple comparisons of differences in the soil test indices, soil respiration and environmental factors under different treatment conditions. Data manipulation and drawing, were performed using Excel 2003 software; all of the statistical analyses were performed with a significance level of 0.05 using StatView 5.0 (SAS Institute, Inc., Cary, NC, USA).

## Results

### Soil properties in response to N deposition

**Fig 2:**
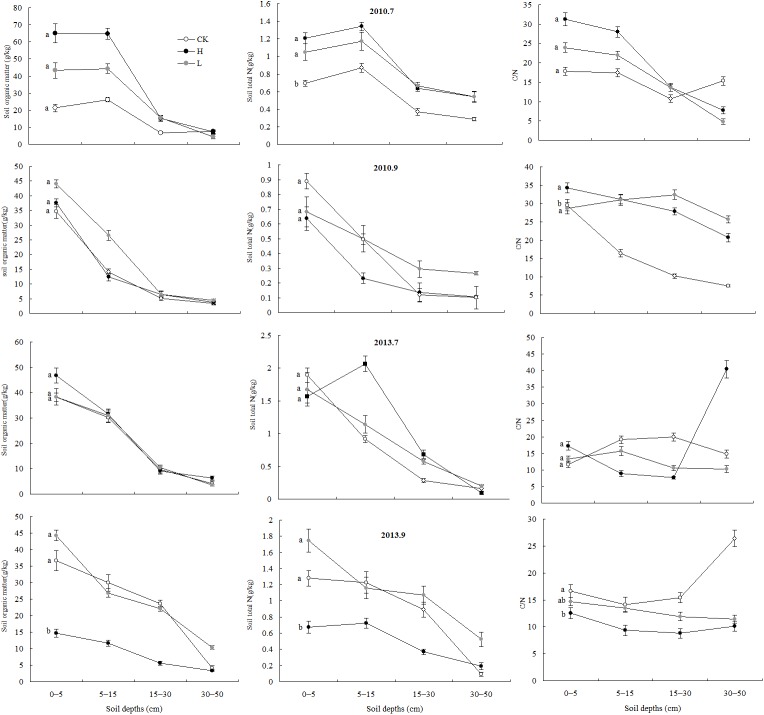
Vertical distribution of soil organic matter, N, and soil C/N ratio for different N treatments (mean ± SD) The lines indicate the vertical distributions of soil organic matter, N, and soil C/N for different levels of N treatment at soil depths of 0–50 cm in July and September 2010 and 2013, and the error bars indicate the standard deviations of the means (n = 3). CK denotes the control treatment (no nitrogen added), L denotes the low-concentration N treatment (37.5 kg·N·ha^-1^·yr^-1^), and H denotes the high-concentration N treatment (112.5 kg·N·ha^-1^·yr^-1^). *P*. *euphratica* samples were selected in an arid area of the Ebinur Lake Area of China.

The soil organic matter content, TN content and C/N ratio at depths of 0–50 cm decreased in the order high-N > low-N > control treatments in July but decreased in the order low-N > high-N > control treatments in September. The soil organic matter content, TN and C/N ratio decreased with increasing depth in the three treatments (Fig 2).

Within 40 months after N deposition (from July 2010 to July 2013), all of the N treatments had increased the amount of soil organic matter, but the influence was not significant (P > 0.05). At 41 months after N deposition (September 2013), the low-N treatment increased the organic matter content, whereas the high-N treatment significantly reduced the organic matter content (P < 0.05) (Table 1). That is, the soil organic matter content significantly decreased over time after high-N treatment, whereas significant changes were not observed over time after the low-N treatment.

All of the N deposition levels clearly increased the TN in the soil within 3 months of N deposition (July 2010); however, for all of the subsequent time points, the TN in the soil was not significantly affected by N deposition. At 41 months (September 2013), the TN in the soil was significantly reduced after high-N deposition and significantly increased after low-N deposition, which suggests that the long-term effects of N deposition on the TN in the soil included a significant reduction after high-N deposition but a significant increase after low-N deposition.

**Table 1:** Comparison of the differences in the soil test index of the 0–50 cm soil layer under the different treatment conditions. Data represent the mean values for soil organic matter, TN, C/N and microorganism quantity for the different treatment conditions (CK, H and L) during the observation period in the study area (July and September of 2009 and 2010). The same lowercase letters in the columns indicate that differences among the indexes were not significant (P > 0.05) for the different months in the same treatment. The same capital letters in the rows indicate that differences among the indices were not significant at P > 0.05 for the different treatments in the same month.

Index	Times	CK	H	L
Organic matter	2010.7	15.478±5.621^aA^	38.005±17.982^aA^	26.913±11.589^aA^
	2010.9	14.359±8.295^aA^	15.073±8.911^abA^	20.378±10.755^aA^
	2013.7	20.567±9.393^aA^	23.405±11.112^abA^	20.800±9.550^aA^
	2013.9	23.594±8.089^aA^	8.813±3.024^bB^	25.890±8.137^aA^
Total N	2010.7	0.555±0.158^aB^	0.933±0.231^abA^	0.857±0.174^aA^
	2010.9	0.435±0.112^aA^	0.277±0.141^bA^	0.401±0.215^bA^
	2013.7	0.814±0.458^aA^	1.102±0.511^aA^	0.896±0.375^aA^
	2013.9	0.872±0.317^aA^	0.490±0.146^abB^	1.126±0.288^aA^
C/N	2010.7	15.345±1.864^aA^	20.159±6.492^aA^	16.058±5.079^bA^
	2010.9	15.904±5.674^aB^	28.488±3.356^aA^	29.443±1.692^aA\^
	2013.7	16.369±2.225^aA^	18.569±8.779^aA^	12.465±1.479^bA^
	2013.9	18.152±3.235^aA^	10.207±0.959^aB^	12.869±0.867^bAB^
Microorganism quantity	2010.7	403675±152807^aA^	486450±207694^aA^	15082±4695^aA^
	2010.9	38094±10339^bA^	8188±1290^bB^	23044±6478^aAB^
	2013.7	432379±110064^aA^	101603±33481^bB^	19351±4788^aB^
	2013.9	75630±31676^bA^	9329±2057^bA^	26853±6688^aA^

In the first year after N deposition, all of the N treatments increased the soil C/N during the peak growing season (July), especially for the last growth stage (September). In the third year after N deposition, the high-N treatment clearly increased the soil C/N ratio, whereas the low-N treatment significantly reduced the soil C/N ratio in July; finally, all N deposition levels significantly reduced the soil C/N ratio in September. Remarkably, the soil C/N ratio in the high-N deposition treatments was higher than that for the low-N deposition treatments in the peak growing season, but this trend was reversed during the latest growth stage.

### Effects of N deposition on soil microbial counts and community structure

**Fig 3:**
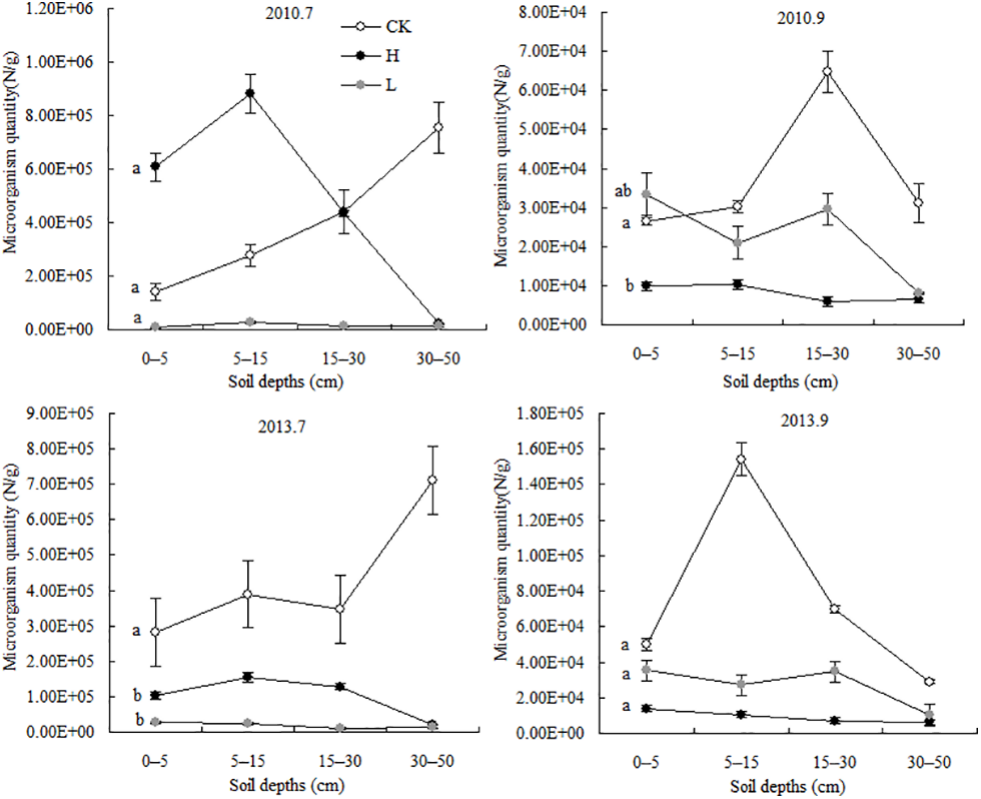
Vertical distribution of soil microorganism quantity after treatment with different levels of N (mean ± SD) The lines show the vertical distribution of soil microorganism quantity for different N treatments at 0–50 cm in July and September in 2010 and 2013, and the error bars indicate the standard deviations of the means (n = 3). The information for each treatment is the same as that in Fig 1.

In July 2010, the soil microbial counts from the different treatments at depths of 0–30 cm decreased in the order high-N > control > low-N treatments. In September 2013, the soil microbial counts on the surface decreased in the order low-N > control > high-N treatments (Fig 3). In all other layers, the counts were highest in the control treatments and then the low-N treatment group, with the lowest counts in the high-N treatments. In July 2013, the counts in all layers decreased in the order control > high-N > low-N treatment. In September 2013, the counts were highest in the control followed by the high-N treatment, with the lowest counts in the low-N treatments. These results suggest that in the early stages of N deposition, microbial counts are increased by high concentrations of N and decreased by low levels of N. However, over longer periods after N deposition treatment, both the low and high levels of N deposition decreased the microbial counts.

The ANOVA results showed that in July 2010 and September 2013, no significant association was observed between the simulated N deposition level and soil microbes. However, in September 2010 and July 2013, the level of N deposition significantly affected the soil microbes (Table 1). These results suggest that in the first year after N deposition, the deposition rate significantly affected the soil microbes in the last growth stage. Over the long term, N deposition rates significantly affected the soil microbes in the peak growing season.

In July 2010 and 2013, fungi and bacteria accounted for a higher total microbial count in the low-N treatment group than in the control and high-N treatment groups. The microbial community structure was similar between the control and high-N groups, in which fungi accounted for the total microbial counts. In September 2010 and 2013, the proportion of fungi and bacteria decreased in the low-N treatment group but increased in the control and high-N treatment groups. The fungi/bacteria ratios in the control, high-N and low-N treatment groups, respectively, were 0.005, 0.003 and 0.050 in July 2010; 0.015, 0.053 and 0.033 in September 2010; 0.007, 0.050 and 0.032 in July 2013; and 0.008, 0.074 and 0.019 in September 2013. These results show that the microbial community structure and function vary with different concentrations of N deposition. Specifically, high and low N deposition induced opposite changes in the microbial community structure (fungi/bacteria ratio), with the former increasing that ratio and the latter reducing it.

### Soil respiration in response to N deposition

#### Soil respiration characteristics in the three treatments

**Fig 4:**
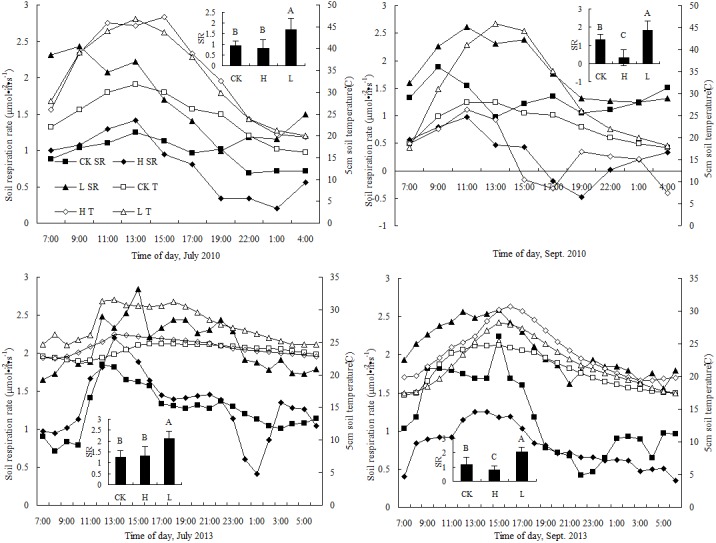
Diurnal variations in different N treatments and their effects on the soil respiration rate and soil temperature at 5 cm The curves in the larger images show the effects of diurnal variations in the different N treatments on the soil respiration rate and temperature at a depth of 5 cm. In the smaller images, the columns indicate the average soil respiration rates for the different N treatments, and the error bars indicate the standard deviations of the means (n = 9). The same capital letters indicate that the difference in soil respiration rate was not significant (P > 0.05) for the different treatments in the same month. *SR*, soil respiration rate (μmol·m^-2^·s^-1^); T, soil temperature (°C) at 5 cm.

As shown in Fig 4, soil respiration varied significantly between day and night for all treatment conditions. Fluctuations were irregular, with peaks generally occurring at 13:00 in July and at 11:00 in September 2010. At the depth of 5 cm, the soil respiration peaks occurred simultaneously with soil temperature peaks, which may have been caused by the increased microbial and plant root activity at higher soil temperatures [31]. The timing of the soil respiration peak varied with the treatment and occurred at 12:00, 13:00 and 14:00 for the control, high-N and low-N treatment groups, respectively. In September 2013, soil respiration and temperature peaked at similar times (12:00, 13:00 and 15:00) under the high-N treatment. For the other treatment groups, the temperature peaks either preceded or lagged behind the soil respiration peaks. In July 2010, soil respiration under the low-N treatment was significantly higher than that under the high-N and control treatments, and the respiration rate in the high-N treatment was slightly but not significantly higher than that in the control treatment. In September 2010, significant differences were observed among the three treatments (P < 0.05), with soil respiration significantly decreasing in the order low-N > control > high-N treatment. In July 2013, soil respiration rates under the low-N and high-N treatments were significantly greater than that under the control treatments but not significantly. In September 2013, soil respiration in the low-N condition was significantly higher than that in the control condition, whereas the soil respiration under the high-N condition was significantly lower than that in the control treatments. As shown in Fig 4, the soil respiration rates varied significantly between the high-N and low-N treatments during the last growth stage (September). However, significant differences were not observed between the control and high-N treatments during the peak growing season (July), suggesting that N deposition had a stronger effect on soil respiration during the last growth stage.

The average changes in the soil respiration rates in the control, high-N and low-N treatments from July 2010 to September 2013 were 1.19±0.39, 0.91±0.51 and 1.99±0.42 μmol·m^-2^·s^-1^, respectively, showing that low N deposition increased soil respiration by approximately 66.7±2.7%, whereas high N deposition reduced soil respiration by approximately 23.6±2.5%.

**Fig 5:**
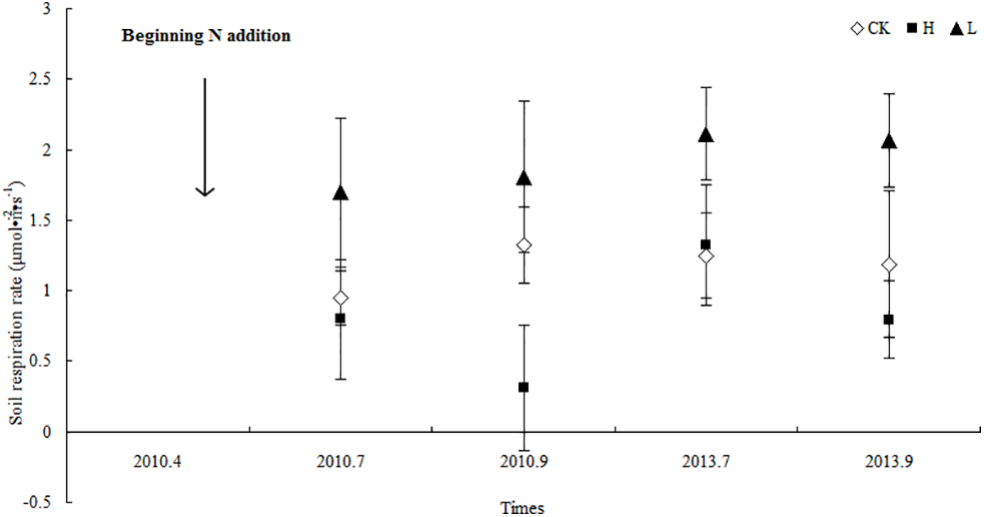
Average soil respiration rate under different treatment conditions (mean ± SD) The N deposition treatment experiment began in April 2010, and the soil respiration rates in fields with different N treatments were measured in July and September of 2010 and 2013. The error bars indicate the standard deviations of the means (n = 9).

Table 2 and Fig 5 illustrate the fluctuations in daily average soil respiration over time under low-N and high-N treatment conditions. The daily average soil respiration under the low-N and high-N treatments peaked in July 2013 (P < 0.05); however, the soil respiration under low-N and high-N deposition conditions at the end of the experiment (September 2013) only slightly exceeded the initial values (P > 0.05). Thus, at approximately 41 months after N deposition, no obvious effects of N fertilizer on soil respiration under the canopy of *P*. *euphratica* were observed.

**Table 2:** Comparison of differences in the soil respiration rates and environmental factors under the different treatment conditions. R*s*, soil respiration rate (μmol·m^-2^·s^-1^); T_-5_, T_-10_, soil temperature (°C) at depths of 5 and 10 cm; W, mean soil moisture of the 0–50 cm layer (%). The same lowercase letters in the columns indicate that differences among the indexes were not significant (P > 0.05) for the different months in the same treatment. The same capital letters in the rows indicate that differences among the indices were not significant at P > 0.05 for the different treatments in the same month.

Treatment	Time	CK	H	L
Rs	2010.7	0.951±0.112^aB^	0.797±0.243^bB^	1.697±0.304^bA^
	2010.9	1.322±0.157^aB^	0.311±0.256^cC^	1.808±0.310^abA^
	2013.7	1.250±0.176^aB^	1.326±0.247^aB^	2.113±0.191^aA^
	2013.9	1.186±0.300^aB^	0.795±0.158^bC^	2.065±0.191^aA^
T_-5_	2010.7	24.420±3.167^aB^	33.860±6.232^aA^	33.470±5.822^aA^
	2010.9	22.920±2.285^aB^	16.535±3.711^cC^	30.130±6.298^aA^
	2013.7	23.750±0.532^aB^	24.279±0.695^bB^	27.702±1.503^abA^
	2013.9	21.000±1.623^aC^	23.700±2.209^bA^	21.900±2.105^bB^
T_-10_	2010.7	24.900±1.597^aB^	29.970±4.032^aA^	29.850±2.442^aA^
	2010.9	22.730±1.573^bB^	16.540±3.196^cC^	26.025±3.179^bA^
	2013.7	22.379±0.153^bC^	22.753±0.232^bB^	25.006±0.686^bA^
	2013.9	20.119±0.808^cB^	22.255±0.839^bA^	20.567±0.609^cC^
W	2010.7	0.160±0.010^aA^	0.139±0.046^aA^	0.150±0.045^aA^
	2010.9	0.128±0.006^bA^	0.145±0.020^aA^	0.172±0.011^aA^
	2013.7	0.103±0.009^cA^	0.135±0.059^aA^	0.146±0.011^aA^
	2013.9	0.117±0.011b^cA^	0.131±0.010^aA^	0.148±0.015^aA^

#### Soil temperature, soil moisture and soil respiration

**Fig 6:**
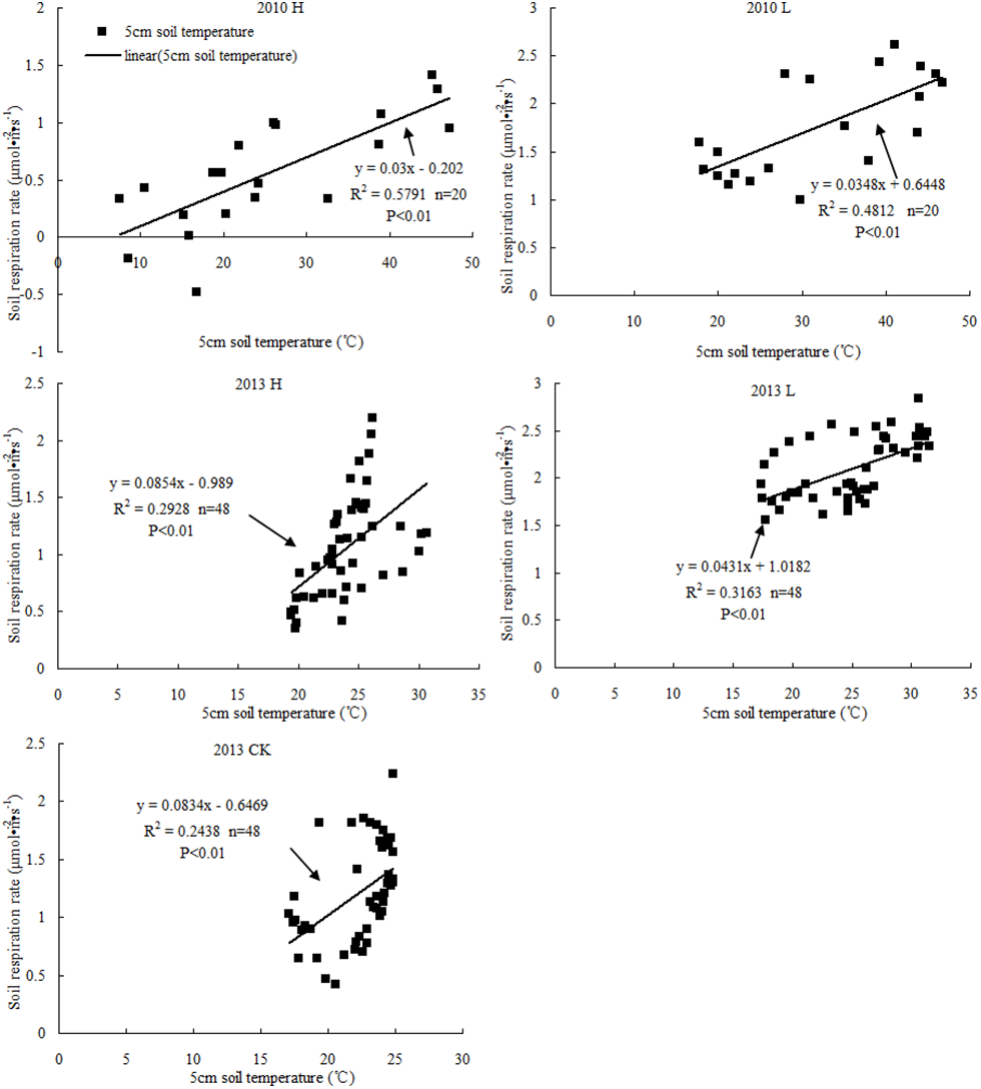
Fitted curve for the soil respiration rate and soil temperature in different treatments Straight lines were used to describe the relationships between the soil respiration value and soil temperature at a depth of 5 cm in different treatment conditions and different years.

N deposition significantly increased the soil temperature at depths of 5 cm and 10 cm (P < 0.05, Table 3) but had little influence on soil moisture (P > 0.05, Table 2). A correlation analysis between soil respiration rate and soil temperature under different treatments using a P-value of 0.05 produced several findings. Under the control treatment, weak positive and negative correlations were observed between the soil temperature and respiration rate. Under the high-N treatment, the soil temperature at all depths were significantly positively correlated with the respiration rates (P < 0.01). Under the low-N treatment, the soil temperature at a depth of 5 cm and the respiration rate were significantly positively correlated (P < 0.05), but at a depth of 10 cm, the positive correlation was weak. Furthermore, the relationship between soil temperature and soil respiration decreased over time after N deposition. The relationship between soil respiration rate and temperature at depths of 5 cm and 10 cm was significant and linear under the high-N treatment (P < 0.05). Under the low-N treatment, a significant exponential relationship was observed between the soil respiration rate and temperature at 5 cm (P < 0.05, Fig 6).

**Table 3:** Relationship between the soil respiration rate and soil temperature under different treatment conditions. T_-5_ and T_-10_ denote the soil temperature (°C) at depths of 5 and 10 cm, respectively. The values denote the correlation coefficient between soil respiration and soil temperature at depths of 5 and 10 cm;

Treatment	Times	Temperature	
		T_-5_	T_-10_
CK	2010	0.252	-0.024
	2013	0.500**	0.104
H	2010	0.761**	0.693**
	2013	0.541**	0.384**
L	2010	0.694**	0.436
	2013	0.562**	0.204

** P < 0.01

In the first year after N deposition, the Q_10_ for soil respiration increased under both the low-N and high-N treatments (Table 4). Compared with the control condition, in the third year after treatment, the high-N treatment increased the soil respiration Q_10_, whereas the low-N treatment reduced the respiration Q_10_. Overall, these results suggest that higher levels of N deposition can increase the temperature sensitivity of soil respiration, whereas lower levels of N deposition reduce the temperature sensitivity of soil respiration.

**Table 4:** Q_10_ values for soil respiration under the different treatment conditions. Q_10_: the temperature sensitivity of soil respiration. Values in the column “Q_10_ value” denote the average Q_10_ for July and September in 2010 and 2013 for each treatment. Values in the “Mean annual value” column denote the average Q_10_ in 2010 and 2013 for each treatment.

Treatment	Time	Q_10_ value	Mean annual value
CK	2010.7	1.448	1.221
	2010.9	0.995	
H	2010.7	1.571	1.799
	2010.9	2.026	
L	2010.7	1.203	1.235
	2010.9	1.266	
CK	2013.7	3.053	2.752
	2013.9	2.452	
H	2013.7	7.668	4.895
	2013.9	2.123	
L	2013.7	1.694	1.492
	2013.9	1.290	

## Discussion

### Effects of N deposition on the physical and chemical properties of soil

Within 40 months after N deposition, the organic content of soil has been shown to increase as N concentrations increase, which may be a result of N promoting above- and below-ground plant growth, resulting in elevated plant root exudates and litter and increased organic matter content [34,44]. However, the variance analysis conducted here shows that significant differences were not observed between the amounts of organic matter in the soil after different levels of N deposition (P > 0.05), which is consistent with the findings of Li [45]. In the 41 months after N deposition, the high-N-deposition treatment significantly reduced the organic content of the soil, whereas significant differences were not observed between the low-N and control treatment conditions. This finding suggests that long-term high-N deposition would result in a “dilution effect” (i.e., when large quantities of NH_4_^+^ are added to the soil, the activity ratio between NH_4_^+^ and other cationic nutrients increases, inhibiting root absorption of other cations because NH_4_^+^ seals the membrane region and prevents other cationic nutrients from adsorbing onto the root membranes), which might lead to soil nutrient imbalances.

Overall, the effect of N deposition on the TN in the soil during the peak growing season and last growth stage varied with the level of N deposition. More specifically, during the peak growing season, the high-N and low-N treatments increased the TN in the soil, and the largest effects were observed for high N deposition (mean total N levels were 1.018 and 0.877 for high and low N deposition, respectively). During the last growth stage, the TN in the soil decreased under the high-N-deposition condition but increased under the low-N-deposition condition, which may have been caused by exogenous N entering the N cycle through incorporation into plant tissues [45]. High N deposition results in increased NO_3_^-^ leaching [46], which reduces the soil N content. The above reasons produced a higher soil C/N ratio under high N deposition than under low N deposition in the peak growth season, with the opposite result in the late growth season. These findings were inconsistent with those reported by Huang [47], who found that N deposition reduced the C/N ratio such that higher levels of deposition corresponded with greater reductions in soil C/N.

### Effects of N deposition on soil microbial counts and structure

Increases in N input can change soil N cycling because N input affects microbial activities involved in litter decomposition, nitrification and denitrification, as well as below-ground ecological processes such as microbial counts, activity and community structure and microorganism-modulated responses to increased N [5,48]. The biological cycles of arid and semiarid ecosystems depend on the ability of soil microorganisms to use pulses of nutrients [49]. N deposition can provide energy and nutrients to microorganisms, thereby increasing their vitality in the short term. These microorganisms are a core component of the biogeochemical cycle and play a vital role in the effects of N deposition on soil structure and soil nutrients, which affect soil respiration.

In this study, only the initial time point (July 2010) showed an increase in microorganism levels with high levels of N deposition, which may have been due to the low levels of soil N restricting increases in the number of microorganisms. This restriction was removed with N deposition, which accelerated the decomposition of organic matter and significantly increased the number of soil microbes [5]. After five months of the N deposition experiment, the microbial counts in the high-N and low-N conditions decreased, perhaps because the long-term N deposition promoted the formation of antagonistic substances and increased the ionic potential of the soil solution. Increasing the ionic potential may reduce the soil’s osmotic potential, thus reducing the water available to the microbial community and the overall microbial count [50–52].

In a temperate broad-leaved/pine forest, Frey et al. [53] showed that N deposition significantly reduced the fungal/bacterial counts, decreasing the decomposition rate and altering the N cycle. Our results showed that N deposition can indeed affect microbial structure and function; however, with increasing time after deposition, the effects of the high and low levels of N deposition on the microbial structural were reversed, with high N deposition increasing and low N deposition decreasing the fungal/bacterial counts. This result may have been caused by changes in the soil properties over time with N deposition.

### Effects of N deposition on soil respiration

#### Differences in soil respiration rates under different treatment conditions

In this study, a low level of N deposition was found to promote soil respiration, whereas a high level of N deposition was found to inhibit soil respiration, indicating that exogenous N input may be closely related to soil respiration responses. Similarly, in the Heshan *Acacia mangium* forest, Cao et al. [25] found that a low level of N deposition promoted soil respiration and high N levels inhibited respiration. The different responses (increases or decreases) of soil respiration to the different levels of N deposition may be associated with the N status of the soil itself [54]. Low-level N deposition may promote soil respiration by increasing the available N and thus removing soil N limitations, which would promote root growth and litter production [44] and increase the soil CO_2_ efflux because high levels of N deposition may elevate the soil N content above the level that is required by plants and microorganisms, thus removing N as a limiting factor. In addition, the increased soil deposition restrains soil respiration [27,54], with the high-N treatment significantly limiting the rate of soil respiration (P < 0.05), which is similar to the results of previous studies [27,55].

Soil respiration consists of three biological processes (plant root respiration, microbial respiration and soil animal respiration) and one chemical oxidation process (organic matter conducted oxidative decomposition followed by CO_2_ release). Increased N deposition enhances soil nitrification by reducing the soil pH, inhibiting plant root growth, reducing root biomass and ultimately limiting plant root respiration [27,55]. N deposition may also inhibit the activity of soil microorganisms and certain soil enzymes (such as lignin-degrading enzymes), thus reducing their capacity to decompose soil organic C [56]. However, sustained soil N deposition may also change the composition of soil animal and microbial communities [53,57,58], restraining their population biomass and decreasing the respiration by soil fauna and microbes. Such an effect would also reduce soil CO_2_ fluxes and eventually decrease the total soil respiration.

### Effects of the sensitivity of soil respiration to temperature

The effects of simulated N deposition on soil respiration and the sensitivity of soil respiration to temperature varied according to location, observation time and overall time since N deposition. Based on data from a deciduous broad-leaved forest of the northern subtropics, Li reported [45] that N deposition increased the soil respiration Q_10_ value but that this increase was not significant. Tu et al. [59] found that N deposition increased the temperature sensitivity of respiration in a *P*. *amarus* forest in a rainy area of western China. However, the same authors found that short-term simulated N deposition did not significantly affect the temperature sensitivity of soil respiration in a *Bambusa pervariabilis* × *Dendrocala mopsi* plantation in rainy western China [60]. In the present study, low levels of N deposition reduced the temperature sensitivity of soil respiration, and this sensitivity was increased when the N levels increased beyond a certain threshold, thus, low levels of N deposition reduced the temperature sensitivity of soil respiration, and this sensitivity was increased when the N levels increased beyond a certain threshold, but this increase or reduce were not significant (P>0.05).

## Conclusions

N deposition indirectly influences soil respiration rates by changing the physical and chemical properties of the soil. N deposition significantly increased the soil temperature but had little effect on the soil moisture. The thermal sensitivity of soil respiration was increased by high levels of N deposition and decreased by low levels of N deposition. All of the tested levels of N deposition affected the microbial counts, structure and function; however, the influence of N deposition was reversed with increasing time after the N treatment. The soil respiration rate presented a different response to N deposition than previously reported for a *P*. *euphratica* community with the same soil background values, in which high N deposition significantly reduced soil respiration and low N deposition significantly increased soil respiration.

## Supporting Information

S1 Appendix Raw data for this study. Soil organic matter, TN, C/N, microorganism quantity, soil moisture, soil temperature and soil respiration rate are shown.
(XLSX)
